# Retinal Pigment Epithelium Expressed Toll-like Receptors and Their Potential Role in Age-Related Macular Degeneration

**DOI:** 10.3390/ijms22168387

**Published:** 2021-08-04

**Authors:** Alexa Klettner, Johann Roider

**Affiliations:** University Medical Center, Department of Ophthalmology, University of Kiel, 24105 Kiel, Germany; Johann.Roider@uksh.de

**Keywords:** retinal pigment epithelium (RPE), toll-like receptors (TLR), age-related macular degeneration (AMD), microglia

## Abstract

(1) Background: Inflammation is a major pathomechanism in the development and progression of age-related macular degeneration (AMD). The retinal pigment epithelium (RPE) may contribute to retinal inflammation via activation of its Toll-like receptors (TLR). TLR are pattern recognition receptors that detect the pathogen- or danger-associated molecular pattern. The involvement of TLR activation in AMD is so far not understood. (2) Methods: We performed a systematic literature research, consulting the National Library of Medicine (PubMed). (3) Results: We identified 106 studies, of which 54 were included in this review. Based on these studies, the current status of TLR in AMD, the effects of TLR in RPE activation and of the interaction of TLR activated RPE with monocytic cells are given, and the potential of TLR activation in RPE as part of the AMD development is discussed. (4) Conclusion: The activation of TLR2, -3, and -4 induces a profound pro-inflammatory response in the RPE that may contribute to (long-term) inflammation by induction of pro-inflammatory cytokines, reducing RPE function and causing RPE cell degeneration, thereby potentially constantly providing new TLR ligands, which could perpetuate and, in the long run, exacerbate the inflammatory response, which may contribute to AMD development. Furthermore, the combined activation of RPE and microglia may exacerbate neurotoxic effects.

## 1. Introduction

Age-related macular degeneration (AMD) is the major cause for blindness and severe visual impairment of the elderly in the industrialized world [1]. It can present in early, intermediate, and two different late forms of the disease, with only the late forms actually threatening vision. A major hallmark of AMD is the appearance of so-called Drusen, subcellular aggregations of lipids, glycoconjugates, and (pro-inflammatory) proteins [2]. The late forms of the disease may present in the “dry” form, in which areas of the retinal pigment epithelium (RPE) degenerate, leaving atrophic patches that may result in geographic atrophy (GA), and the exudative or wet form of the late disease, in which vessels grow from the choroid into and beneath the retina (choroidal neovascularization, CNV), which are immature and leaky, causing edema and tissue destruction [3,4]. So far, treatment is only available for the exudative forms of the disease, targeting the vascular endothelial growth factor (VEGF) [5]. The untreated, exudative AMD may progress to a subretinal fibrosis and a fibrotic scar [6]. AMD is a multifactorial disease with different factors contributing to its onset and progression. While age, genetic disposition (mainly concerning genes of the complement system), and environmental factors contribute to the risk of developing AMD, on the tissue level, oxidative stress, lipid dysregulation, and angiogenic signaling are implicated as major pathogenic factors [7,8,9]. Furthermore, inflammation, especially low-grade chronic inflammation, is regarded as a major factor in AMD development [10,11,12].

On a cellular level, the pathogenesis of the disease is happening at the photoreceptor/retinal pigment epithelium (RPE)/choroid complex [13], with the RPE generally considered to be the primary contributor to disease development [14,15]. The RPE is a single-layered epithelium, situated between the choroid and the photoreceptors. It has many functions to support the photoreceptor cells and maintain vision. It makes up the outer blood-retinal barrier, controlling the entry into the outer retina and supplying the photoreceptor cells with nutrients from the choroid. Additionally, it facilitates waste disposal in the opposite direction as well as secretes a variety of cytokines, such as VEGF, which protect the photoreceptors and the choroidal endothelium. Moreover, it takes up shed photoreceptor outer segments and takes part in recycling the photopigment [16]. It protects the photoreceptors by defending the tissue against oxidative stress [17] and helps keep up the immune privilege [18]. The RPE is a major contributor to the blood-retinal barrier [19]. Furthermore, it contributes to an anti-inflammatory milieu by secreting anti-inflammatory cytokines and suppresses T-cell activation by inducing anergy, apoptosis or regulatory T-cell differentiation [20,21,22,23,24]. However, the RPE is also involved in the inflammatory response in the retina. The RPE can act as a sentinel, being strategically located at the interface between the blood supply (choroid) and the photoreceptors [13], and is equipped with Toll-like receptors (TLR) [25].

Toll-like receptors are pattern recognition receptors, designed to detect potential dangerous molecules, either pathogen-associated molecular patterns (PAMPS), e.g., lipopolysaccharid from Gram-negative bacteria or danger-associated molecular patterns (DAMPS), e.g., RNA from dying cells [26,27]. In humans, 10 different TLR have been described [28], with TLR2, -3, and -4 being of the highest interest in regards to the RPE and AMD. TLR can be located on the cell surface, e.g., TLR2 and TLR4 or intracellularly localized, as TLR3, which can be found in the endosome [29]. Each TLR detects specific patterns, with TLR2 (together with TLR1 or TLR6) recognizing, among others, peptidoglycans as indicators of Gram-positive bacteria [30], TLR3 detecting double strand RNA as found in viruses and dying cells [31], and TLR4 detecting lipopolysaccharide (LPS), as an indicator for Gram-negative bacteria [27]. TLR are transmembrane proteins with extracellular leucine-rich repeats to bind the PAMPS and DAMPS and an intracellular TIR (Toll-interleukin-1 receptor) domain, responsible for signal transduction [29]. Activation and signaling pathways are dependent on the respective TLR and ligand, and generally result in the expression of inflammatory cytokines and are often being mediated via nuclear factor ‘kappa-light-chain-enhancer’ of activated B-cells (NFκB) [29]. Excellent reviews are available on the different signal transduction pathways and regulators [28,29].

TLR are widely expressed in the eye [32,33,34], playing a role in a variety of ophthalmological conditions such as corneal immunity and inflammation [35,36,37], conjunctivitis [34,38] or protection from endophthalmitis [39,40]. The aim of this review is to give an overview on the recent knowledge regarding TLR involvement in AMD, its activation in the RPE, and the implications this might have for AMD pathophysiology.

## 2. Results

### 2.1. Toll-Like Receptors in AMD

#### 2.1.1. TLR Polymorphisms and Patient Studies

The influence of TLRs on the pathophysiology of the AMD is complex. The literature is filled with contradicting reports on the association of TLR polymorphisms and AMD, which have been rather recently reviewed in [41]. While some reports see clear, sometimes protective associations of certain TLR gene variants, others see little or no association at all. The differences are likely to be associated with the genetic background of the investigated populations (e.g., American Caucasians vs. Han Chinese population), with the respective single nucleotide polymorphism (SNP) of the TLR, low frequencies of the investigated alleles, but also with the different subtypes of AMD.

Little has been published on the potential involvement of TLR2 SNPs in regards to AMD. In one study conducted in Turkey, the TLR2 Arg753Gln genotype had approximately four times greater risk of AMD compared with the TLR2 Arg753Arg genotype [42].

For TLR3, the published results are diverse. A protective effect of a specific polymorphism in TLR3 (L412F variant, rs3775291) has been shown in geographic atrophy, but not with early AMD or CNV, as shown in a study in Caucasian Americans from three different regions (Utah, Maryland, Oregon) [43]. However, several authors expressed their concerns about the study, questioning its conclusions [44,45,46,47]. In addition, other studies could not replicate this result [48,49,50]. In a meta-analysis, however, Zhou et al. could confirm the association between this variant and GA. As a reason for the lack of association in the previous studies, they discussed small sample size, stratification, and heterogeneous study populations [51]. Furthermore, they showed that the L412F variant did not alter the expression of TLR3, but decreased its binding capacity to dsRNA and consequently shows a reduced activation of NFκB after stimulation [51]. While this association may be of importance for Caucasian subpopulations, no association has been found in a study with Indian subjects [52], supported by another meta-analysis, which showed an association with Caucasian, but not Asian subjects [53]. Moreover, no association between the polymorphism of TLR3 has been drawn to the neovascular subtype of AMD, as shown for Chinese study populations [54,55] or with the growth of GA in AMD patients treated with anti-VEGF compounds (American study population of 43 different centers) [56]. In an exhaustive study investigating 68 SNPs of several TLRs, associations of some SNPs, e.g., for TLR3 and TLR7 were shown to be statistically significant in one cohort of patients (Dallas cohort), but not in another cohort (Michigan cohort) [48].

The opposite effects of a SNP in a coding region of TLR4 have been studied, with TLR4 variant D299G (rs4986790) being associated with susceptibility to AMD in one study (in Caucasian subjects) [57], but not in follow-up studies (in Indian, Turkish, and Caucasian subjects) [42,43,49,58,59]. However, two recent meta-analyses did find a higher risk associated with this SNP [60,61]. In a recent Chinese study, an association with an SNP in a non-coding region of TLR4 with AMD has been shown [62].

Taken together, the evidence of a genetic TLR involvement in AMD is not as strong as, e.g., shown for the complement factor (CF) H polymorphisms, but there might be some associations with the AMD development, depending on the genetic background of the carrier.

In addition, in a small study in a cohort in China, it was shown that blood mononuclear cells (PBMC) from patients with wet AMD expressed more TLR2 and TLR3 (but not TLR1 or TLR4-10). Furthermore, these cells displayed an elevated secretion of pro-inflammatory cytokines when treated with TLR2 or TLR3 agonists [63]. These data indicate that extra-ocular TLR activation may be involved in AMD development. In CNV membranes from patients with wet AMD, the local expression of TLR3 was also elevated (in the RPE) [64].

#### 2.1.2. Pre-Clinical Data

Apart from clinical data, mouse models have given an indication of potential TLR involvement in AMD development. In a laser-induced CNV model, TLR2 activation (by *Chlamydia pneumoniae*) elevated interleukin (IL)-6 and VEGF and exacerbated neovascularization [65]. This is of special interest, as both the retinal pigment epithelium and peripheral macrophages could contribute to this CNV by enhanced cytokine secretion [63,65]. Indeed, in addition to increasing the CNV area, TLR2 activation increased the infiltration of monocytic cells (microglia, macrophages) and neutrophils in a laser-induced CNV mouse model [66]. TLR2 activation by ω-(2-carboxyethyl)pyrrole (CEP), a product of lipid oxidation, can exert pro-angiogenic effects, shown for various tissues [67] and is elevated in plasma and retina of AMD patients [68,69]. Furthermore, in mouse models of oxidative stress, including CEP-induced retinal degeneration, TLR2 inhibition resulted in decreased complement deposition and activation, and a protection of the RPE and photoreceptors from cell death. In addition, TLR2 inhibition reduced the migration of microglia or macrophages into the subretinal space [70]. Of note, the TLR2 activating and pro-angiogenic properties of CEP have been challenged [71] and CEP has been suggested to enhance TLR2(/TLR1) activation by specific agonists [72]. In addition, TLR2 activation has been shown to be involved in spontaneous CNV development in a mouse model [73].

Activation of TLR3 has been shown to elicit cell death in different retinal cells, such as photoreceptors [74], retinal ganglion cells [75], and retinal pigment epithelium cells [76,77] (see below). TLR3 has been implicated to be responsible for retinal degeneration in a mouse model of cone-rod dystrophy as well as in light-induced degeneration [76]. However, it has been shown that when activated under oxidative stress, TLR3 activation may actually protect photoreceptors [78] or retinal pigment epithelial cells [79]. In addition, activation of TLR3 has been shown to prevent angiogenesis in a laser-induced CNV mouse model [80].

Various Drusen components have been suggested to activate TLR4 signaling. Amyloid ß is a constituent of Drusen and has been indicated to be involved in AMD pathogenesis [81]. In cell culture experiments, it has been shown that amyloid ß induces the secretion of pro-inflammatory and pro-angiogenic cytokines in RPE cells and induces tube formation, an indicator of angiogenesis, via the activation of TLR4 [82]. Similarly, 7-Ketocholesterol, a oxidized cholesterol product, which is a constituent of Drusen and has been implicated in AMD development [83], induces pro-inflammatory cytokine expression via TLR4 signaling [84]. In addition, in mouse models of retinal degeneration, TLR4 activation by photoreceptor proteins contributed to an increase in subretinal monocytes/microglia, pro-inflammatory cytokine production, and retinal degeneration [85].

Complement is considered to be a major contributor to AMD pathogenesis, and the systemic activation of TLR2, -3, and -4 has been indicated to increase the expression and activation of the alternative complement pathway (C3, CFB) in mouse eye tissue. However, it has to be pointed out that whole eye homogenates were used in this study. Therefore, the exact location or the biological significance of this finding is not known [86]. Subretinal fibrosis is a late-stage after subretinal hemorrhage in late AMD. In a mouse model of subretinal fibrosis, TLR2 and TLR4 were shown to reduce subretinal fibrosis [87]. Taken together, the preclinical data indicate that TLR is involved in a variety of pathophysiological pathways associated with AMD development.

### 2.2. Toll-Like Receptors in the RPE

#### 2.2.1. TLR Expression in the RPE

RPE cells have been shown to express various TLRs (1–7, 9, 10) with TLR3 considered to be the most abundant [88]. Expression of TLRs in the RPE can be elevated by pro-inflammatory stimuli, such as polyinosinic:polycytidylic acid (Poly I:C), as an agonist of TLR3 or interferon (IFN) γ [88,89,90]. Interestingly, the effect is not limited to the activated TLR, as Poly I:C increased the expression of TLR2, TLR3, and TLR4 in the RPE [88]. In addition, TLR2 expression can be enhanced by LPS, chemical TLR2 agonists, Poly I:C or *Chlamydia pneumoniae* [65,73,88,91].

Of note, in the widely used RPE cell line ARPE-19, the expression of TLR3 and TLR4 has been shown [76]. However, ARPE-19 has been described as not responding to LPS due to the lack of other appropriate components for TLR4 activation (MD-2, CD14) [84], while other authors find that ARPE-19 responds to LPS [92]. Amyloid ß has been shown to elevate the expression of TLR4 in ARPE-19. In addition, it induced the elevation of secretion of IL-6, IL-8, IL-33, and VEGF via TLR4 signaling [44]. Moreover, the complement factor C5a increased the expression of TLR4 in ARPE-19 cells [93].

#### 2.2.2. TLR Activation in the RPE

The activation of TLR in the RPE primarily induces pro-inflammatory cytokine secretion, which depends on the stimulus, time frame of stimulation, and TLR targeted. The activation of TLR2 by *Chlamydia pneumoniae* induces the secretion of IL-6 and VEGF but not tumor necrosis factor (TNF) α [65]. In addition, TLR2 activation can interfere with RPE tight junctions, resulting in decreased expression or translocation of tight junction proteins, and impairing their barrier function [94]. Moreover, an increased expression of complement factors CFB and C3 has been shown after TLR2 activation [70]. The stimulation of TLR2 with the synthetic TLR2 agonist PAM2CSK4 induces the secretion of IL-6, IL-1ß, IL-8, monocyte chemoattractant protein (MCP) 1, and TNFα in RPE cells [73,94,95]. When tested in RPE/choroid organ cultures allowing for a separation of apical (towards the retina) and basal (towards the choroid) secretion, IL-1ß and TNFα were secreted almost exclusively on the basal side, while IL-6 secretion could be seen basally and, to a lesser degree, apically [94].

RPE cells constitutively express TLR3, as shown for human and porcine RPE [77,88,96]. In fact, TLR3 is the most abundantly expressed TLR in the RPE [88], and its elevation can be enhanced by activation [88]. Stimulation with the TLR3 agonist Poly I:C [31] induced the expression and/or secretion of IFN-γ (but not IFN-α), IL-6, IL-1ß, IL-8, TNFα, MCP-1, and soluble intercellular adhesion molecule (sICAM)-1 [88,91,94,95,97] as well as the expression of hypoxia-inducible factor (HIF)-1α, junctional adhesion molecule (Jam)-1, ICAM-1, and basic fibroblast growth factor (bFGF) [25,90,91]. Moreover, an induction of genes of the complement system (C5, C9, CFH, CFB) has been described [91]. When tested in RPE/choroid organ cultures allowing for a separation of apical and basal secretion, IL-1ß and TNFα were secreted almost exclusively on the basal side, while IL-6 secretion could be seen basally and, to a lesser degree, apically [94]. In addition, TLR3 activation can induce VEGF secretion, however, this may be a concentration dependent effect [77,90]. Moreover, TLR3 activation may induce the activation of Mitogen-activated protein kinases (MAPK; extracellular signal-regulated kinase (ERK) 1/2, p38, c-Jun N-terminal kinase (JNK)) [77,91]. Concerning the RPE function, the activation of TLR3 hardly affects RPE phagocytosis [25,97], but may reduce the RPE barrier function [94].

In addition to its pro-inflammatory effects, TLR3 activation can induce cell death in RPE cells [43,76,77,79,97], which may be executed by (programmed) necrotic [74] or apoptotic pathways [76]. The pathway of cell death may be concentration dependent, as, e.g., cell death induced by 10 µg/mL but not by 100 µg/mL was mediated by JNK MAPK [77]. Interestingly, in the presence of oxidative stress, TLR3 activation confers a protective effect in RPE cells, both shown for primary (murine) RPE cells and ARPE-19. This protection is mediated via the activation of the transcription factor signal transducer and activator of transcription (STAT) 3 [78].

As pointed out above, RPE cells express TLR4 (and CD14, as a co-receptor for LPS detection), and its expression can be enhanced by LPS stimulation [98]. Stimulation of TLR4 with LPS induces the expression and secretion of IL-8, IL-6, TNFα, and IL-1ß [94,97,98,99] and the expression of cyclooxygenase (COX)-2 and inducible nitric oxide synthase (iNOS) [99]. When tested in RPE/choroid organ cultures, IL-1ß and TNFα were secreted almost exclusively on the basal side, while IL-6 secretion was mainly basally found [94]. TLR4 mediated secretion of IL-6 and IL-8 could be elevated by the complement factor C5a [93]. Regarding the RPE function, TLR4 stimulation with LPS also interferes with the barrier function of RPE cells, as shown for primary RPE, RPE/choroid explants, and ARPE-19 cells [94,99]. Moreover, TLR4 stimulation with LPS can reduce the cell viability and phagocytic activity of primary RPE cells [97]. Of high interest, the long-term stimulation with LPS reduced the expression of RPE65, an enzyme important for the recycling of the visual pigment [97]. These data strongly indicate that, in addition to inducing pro-inflammatory cytokines, prolonged TLR4 activation may interfere with the RPE function. In addition to inflammation, TLR4 has additional tasks in the RPE, as it participates in the recognition and phagocytosis of photoreceptor outer segments [100].

The activation of TLR9 in the RPE induces the secretion of IL-8 (but not MCP-1) in RPE cells (shown for ARPE-19) [25]. Furthermore, the activation of TLR9 increased phagocytic activity in RPE cells (shown for ARPE-19) [25]. An overview of the effects of TLR activation in RPE cells can be found in Table 1.

#### 2.2.3. TLR-Activated RPE and Microglia/Monocytes

The innate immunity of the retina is mainly mediated by the retina-specific cells of the monocytic lineage, the microglia, which has also been implicated in contributing to various retinal diseases [101]. Several lines of studies have indicated that the activated RPE and microglia interact in inflammatory signaling. In addition, the RPE can interact with monocytes derived from the blood.

TLR2 activation in RPE results in a polarized apical secretion of MCP-1, a chemokine that attracts mononuclear cells [70]. Concomitantly, TLR2 activation has been indicated to attract macrophages to CNV lesions (in mouse models) and its inhibition results in a reduced number of macrophages and a reduced CNV in these models [73]. In addition, TLR2 activation in RPE cells reduces the secretion of IL-8 and TNFα, but increases the secretion of IL-1ß (in higher concentrations) in microglia [95].

The activation of TLR3 in the RPE promotes the chemotaxis and adhesion of monocytic cells [25,96]. In addition, TLR3-activated RPE cells inhibit the pro-inflammatory activation of monocytes by reducing the expression of COX-2 and iNOS [96]. Furthermore, they induce the expression of Fas ligand (FasL), which may contribute to an anti-angiogenic phenotype [96,102]. RPE cells also interfere with the pro-inflammatory activity of microglia cells, including the expression and secretion of proteins. TLR3 stimulated RPE cells show an inhibitory effect of iNOS expression in microglia cells [103]. Moreover, TLR3 stimulated RPE cells reduced the secretion of IL-8 and TNFα in microglia cells, while IL-6 secretion in microglia cells showed some induction and IL-1ß was not changed [95]. However, on an mRNA level, both IL-6 and IL-1ß had been elevated [103]. Furthermore, the expression of COX-2 in the microglia was also elevated by RPE cells [103]. Conversely, TLR3 activated RPE cells do not change the phagocytic activity of microglia [103].

TLR4 stimulated RPE cells show an inhibitory effect of iNOS expression in microglia cells [95]. They also reduced the secretion of IL-8 and TNFα in microglia cells, and a reduction of IL-6 mRNA expression could be found [95]. The mRNA expression and cytokine secretion of IL-1ß was not changed by TLR4 stimulated RPE cells [95].

Of interest, irrespective of the regulating effects of RPE on microglia activation, microglia cells that were treated with the supernatant of TLR-activated RPE cells induced cell death in a neuronal cell line [95].

Taken together, the studies indicate a differentiated influence of TLR activated RPE cells on (retinal) microglia and (blood-derived) monocytes. Interestingly, while the RPE itself reacts with a pro-inflammatory cytokine release, it shows a differentiated effect on microglia by “fine-tuning” their cytokine release, reducing IL-8 (which is considered pro-angiogenic [104]) and TNFα (which is considered neurotoxic [105]). Still, the expression of the pro-inflammatory enzyme COX-2 was enhanced in microglia by TLR-activated RPE, in a clear distinction to monocytes, where TLR-activated RPE reduced the expression of COX-2 (and iNOS). In addition, TLR stimulated RPE increased the neurotoxicity of activated microglia cells.

### 2.3. Potential Role of TLR Activation of the RPE in the Development of AMD

**Figure 1:**
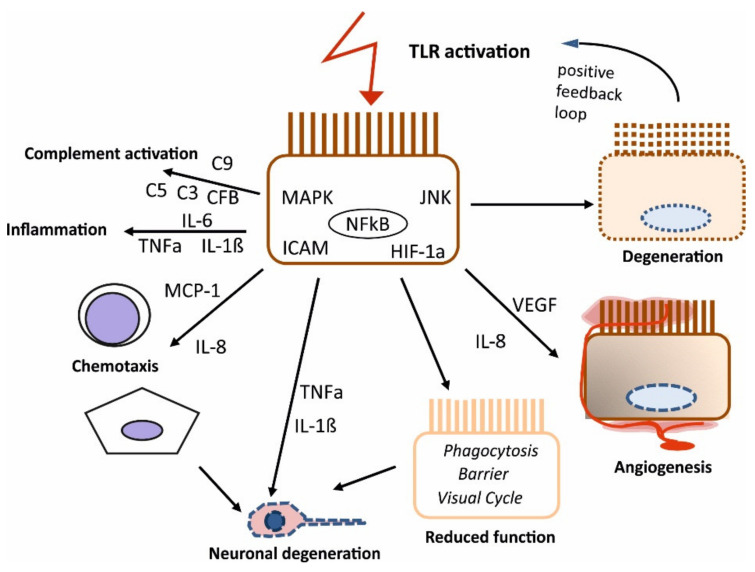
Schematic effects of summarized TLR activation in the RPE and its potential contribution to AMD development. Activation of TLR (summarized for TLR2, -3, and -4) induces the activation and expression of various genes and cytokines, potentially inducing degeneration, angiogenesis, reduced RPE function, chemotaxis of mononuclear cells (monocytes (round), microglia (star shaped)), neuronal degeneration, pro-inflammatory cytokine release, and complement activation.

Taken together, the published data clearly show a strong involvement of TLR activation in pro-inflammatory cytokine secretion in the RPE, indicating their role as a sentinel and active contributor to the innate immunity of the retina. The pro-inflammatory cytokines induced by TLR activation have been implicated in AMD development (IL-6, IL-8, MCP-1 [106,107]), and may be involved in AMD pathogenesis. Of interest is the polarized reaction of the RPE to TLR stimulation, indicating a differentiated response of the RPE in the retinal and choroidal direction [94]. Moreover, pro-angiogenic cytokines induced by TLR activation (VEGF, IL-8) may contribute to the development of CNV. The activation of the TLR in the development of AMD could be mediated by pathogens, such as *Chlamydia pneumoniae* or by degenerating retinal cells [26,65]. As TLR activation, especially considering TLR3, may induce cell death in the retina, e.g., in the RPE and endothelial cells [77,108], this could perpetuate the situation, leading to a vicious cycle of activation of TLR, pro-inflammatory signaling, cell death, and more activation of TLR by the dying cells. However, differences between the activation of different TLRs have to be considered, as well as the difference between short-term (acute) and long-term (chronic) effects. The activation of TLR3 may be of higher significance for long-term induced cell death and the consequent potential vicious cycle than the activation of TLR4, as it was recently shown that Poly I:C induces a severe decrease in RPE cell viability over the course of an activation of 4 weeks, while the toxic effect of LPS was only seen in short (24 h) and medium (7 days) stimulation [97]. Concerning pro-inflammatory cytokine secretion, both stimulation of TLR3 and TLR4 show a similar reaction pattern, with IL-1ß only and IL-6 mainly acutely induced, while IL-8 is elevated both acutely and chronically (investigated for 4 weeks), again stressing its potential involvement in neovascularization [97]. In addition, TLR activation can contribute to AMD development by interfering with the RPE barrier function [94], and, especially in long-term stimulation, with protein expression, as shown for long-term stimulation with LPS and the visual cycle protein RPE65 [97]. On the other hand, the TLR activation of RPE cells may exert a certain calming effect on monocytic cells [96], with an anti-inflammatory and potentially cell-death inducing effect on monocytes and an activating but regulating effect on the pro-inflammatory reaction of microglia, at least in acute stimulation [95,103]. However, TLR-stimulated RPE may increase the neurotoxicity of microglia, which also may indicate an involvement in the development of AMD [95]. However, it must be stressed that these data were obtained in vitro, therefore its relevance in vivo needs to be confirmed. It would also be of interest to investigate how long-term TLR activated RPE would influence monocytic cells, both considering pro-inflammatory activation and neurotoxicity. A schematic of the influence of TLR activation on the RPE which is of potential impact for AMD development is depicted in Figure 1.

Further research is needed to elucidate the role of TLR in the development of AMD, concerning both in vitro and in vivo models. The consequence of activation of TLR in the RPE and its interaction with the cells of the immune system needs to be investigated further. In addition, the role of TLR in the development of AMD should be investigated in animal models mimicking the development of age-related changes in the retina [109,110,111]. Finally, their role in the human situation need to be further investigated. While intervention studies have to be regarded premature considering the current studies and available data, the analysis of donor eyes and tissues of AMD patients regarding TLR expression and activation, as well as identification of the present DAMPS and PAMPS would strongly increase our knowledge and pave the way for potential interventional studies. Understanding the precise contribution of the different TLR to AMD development and identifying the precise activators and contributing pathways could lead to new avenues for (early) AMD prevention.

## 3. Methods

In this systematic review, publications concerning TLR and RPE in AMD related research have been presented. Suitable publications have been searched in PubMed (National Library of Medicine), using the following search terms: Retinal pigment epithelium AND age-related macular degeneration AND Toll-like receptors (21 hits), retinal pigment epithelium AND Toll-like-receptor (41 hits); Toll-like receptors AND age-related macular degeneration (87 hits); without doublings, a total of 106 publications were found of which 54 were included in this study. Only studies published in the English language were considered. In addition, in subsequent searches, five additional papers were included in the manuscript which were not found in the original search. A list of the included studies can be found in Supplementary Table S1.

## 4. Conclusions

The role of TLR in the development of AMD is not sufficiently elucidated yet. While a direct effect of TLR SNP is controversially discussed, pre-clinical data indicate a role of TLR activation in AMD pathogenesis. Especially the activation of TLR in RPE cells, namely, TLR2, -3, and -4 induces a profound pro-inflammatory response that may contribute to (long-term) inflammation by induction of pro-inflammatory cytokines and by causing RPE cell degeneration, constantly providing new TLR ligands and thereby perpetuating and, in the long run, exacerbating the inflammatory response.

## Figures and Tables

**Table 1:** Effects of TLR activation in RPE cells.

TLR	Agonist	Effect	Reference
TLR2	*Chlamydia pneumonia*	Secretion IL-6, VEGF	[65]
		Expression MCP-1, IL-1ß, IL-8	[73]
	PAM2CSK4	Expression CFB, C3	[70]
		Expression/secretion IL-6, IL-1ß, IL-8, MCP1, TNFα	[73,94,95]
		Decreased barrier function, reduced expression tight junction	[94]
TLR3	Poly I:C	Expression/secretion IL-6, IL-1ß, IL-8, TNFα, MCP-1, sICAM-1, IFN-ß	[25,88,90,91,94,95,97]
		Expression HIF-1α, Jam-1, ICAM-1, bFGF, C5, C9, CFB	[25,90,91]
		VEGF	[77]
		Activation of ERK1/2, p38, JNK	[77,91]
		Reduced barrier function	[94]
		Induced cell death	[43,74,76,77,97]
	Poly I:C + paraquat	Protection from oxidative stress	[78,79]
TLR4	LPS	Expression/secretion IL-8, IL-6, TNFα, IL-1ß	[94,97,98,99]
		Expression COX-2, iNOS	[99]
		Reduced barrier function	[94,99]
		Reduced cell viability	[97]
		Reduced phagocytosis	[97]
		Reduced expression RPE65	[97]
TLR9	CpG-DNA	Secretion IL-8	[25]
		Increased phagocytosis	[25]

## Supplementary Materials

The following are available online at https://www.mdpi.com/article/10.3390/ijms22168387/s1.
