# Supplementary material for: Retinal Pigment Epithelium Expressed Toll-like Receptors and Their Potential Role in Age-Related Macular Degeneration
Source: Int J Mol Sci. 2021 Aug 4;22(16):8387. doi: 10.3390/ijms22168387 (PMC8395065; doi:10.3390/ijms22168387)
Supplement: Supplementary file 1 [file ijms-22-08387-s001.zip › ijms-1297953-supplementary.pdf]

Supplemental

Table S1. List of included studies

Authors	Titel	Journal
Allikmets et al.	Geographic atrophy in age-related macular degeneration and TLR3.	N Engl J Med. 2009 May 21;360(21):2252-4.
Brosig et al.	Gene expression regulation in retinal pigment epithelial cells induced by viral RNA and viral/bacterial DNA.	Mol Vis. 2015 Aug 31;21:1000-16.
Chen et al.	Wogonin protects human retinal pigment epithelium cells from LPS-induced barrier dysfunction and inflammatory responses by regulating the TLR4/NF-kappaB signaling pathway.	Mol Med Rep. 2017 Apr;15(4):2289-2295.
Chen et al.	TLR4 inhibitor attenuates amyloid-beta-induced angiogenic and inflammatory factors in ARPE-19 cells: Implications for age-related macular degeneration.	Mol Med Rep. 2016 Apr;13(4):3249-56.
Chintala et al.	Activation of TLR3 promotes the degeneration of retinal ganglion cells by upregulating the protein levels of JNK3.	Invest Ophthalmol Vis Sci. 2015 Jan 6;56(1):505-14.
Cho et al.	Toll-like receptor polymorphisms and age-related macular degeneration: replication in three case-control samples.	Invest Ophthalmol Vis Sci. 2009 Dec;50(12):5614-8.
Crowley et al.	Induction of Ocular Complement Activation by Inflammatory Stimuli and Intraocular Inhibition of Complement Factor D in Animal Models.	Invest Ophthalmol Vis Sci. 2018 Feb 1;59(2):940-951.
Cheng et al.	Toll-like receptor 3 polymorphism is not associated with neovascular age-related	Genet Mol Res. 2014 Jan 17;13(1):302-9.

	macular degeneration and polypoidal choroidal vasculopathy in the Chinese.	
Despriet et al.	Comprehensive analysis of the candidate genes CCL2, CCR2, and TLR4 in age-related macular degeneration.	Invest Ophthalmol Vis Sci. 2008 Jan;49(1):364-71.
Dietrich et al.	Interaction of inflammatorily activated retinal pigment epithelium with retinal microglia and neuronal cells.	Exp Eye Res. 2020 Oct;199:108167.
Duncan et al.	Repeat exposure to polyinosinic:polycytidylic acid induces TLR3 expression via JAK-STAT signaling and synergistically potentiates NFκB-RelA signaling in ARPE-19 cells.	Cell Signal. 2020 Feb;66:109494.
Ebihara et al.	Distinct functions between toll-like receptors 3 and 9 in retinal pigment epithelial cells	Ophthalmic Res. 2007;39(3):155-63.
Edwards et al.	Toll-like receptor polymorphisms and age-related macular degeneration.	Invest Ophthalmol Vis Sci. 2008 Apr;49(4):1652-9.
Edwards et al.	Geographic atrophy in age-related macular degeneration and TLR3.	N Engl J Med. 2009 May 21;360(21):2254-5.
Elner et al.	TLR4 mediates human retinal pigment epithelial endotoxin binding and cytokine expression	Trans Am Ophthalmol Soc . 2005;103:126-35.
Feng et al.	A Proinflammatory Function of Toll-Like Receptor 2 in the Retinal Pigment Epithelium as a Novel Target for Reducing Choroidal Neovascularization in Age-Related Macular Degeneration.	Am J Pathol. 2017 Oct;187(10):2208-2221.
Fujimoto et al.	Choroidal neovascularization enhanced by Chlamydia pneumoniae via Toll-like receptor 2 in the retinal pigment	Invest Ophthalmol Vis Sci. 2010 Sep;51(9):4694-702.

	epithelium.	
Gounarides et al.	Lack of involvement of CEP adducts in TLR activation and in angiogenesis.	PLoS One. 2014 Oct 24;9(10):e111472.
Güven et al.	Toll-Like Receptors 2 and 4 Polymorphisms in Age-Related Macular Degeneration.	Curr Eye Res. 2016 Jun;41(6):856-61.
Grunwald et.	Growth of geographic atrophy in the comparison of age-related macular degeneration treatments trials.	Ophthalmology. 2015 Apr;122(4):809-16
Hettich et al.	The retinal pigment epithelium (RPE) induces FasL and reduces iNOS and Cox2 in primary monocytes.	Graefes Arch Clin Exp Ophthalmol. 2014 Nov;252(11):1747-54.
Huang et al.	7-Ketocholesterol-induced inflammation signals mostly through the TLR4 receptor both in vitro and in vivo.	PLoS One. 2014 Jul 18;9(7):e100985.
Kaur et al.	Analysis of CFH, TLR4, and APOE polymorphism in India suggests the Tyr402His variant of CFH to be a global marker for age-related macular degeneration.	Invest Ophthalmol Vis Sci. 2006 Sep;47(9):3729-35.
Klein et al.	Progression of geographic atrophy and genotype in age-related macular degeneration.	J. Ophthalmology. 2010 Aug;117(8):1554-9, 1559.e1
Kleinman et al.	Sequence- and target-independent angiogenesis suppression by siRNA via TLR3.	Nature. 2008 Apr 3;452(7187):591-7.
Kleinman et al.	Short-interfering RNAs induce retinal degeneration via TLR3 and IRF3.	J. Mol Ther. 2012 Jan;20(1):101-8.
Klettner et al.	Toll-like receptor 3 activation in retinal pigment epithelium cells - Mitogen-activated protein kinase pathways of cell death and vascular endothelial growth factor secretion.	Acta Ophthalmol. 2013 May;91(3):e211-8

Klettner et al.	Retinal pigment epithelium cells alter the pro-inflammatory response of retinal microglia to TLR-3 stimulation.	Acta Ophthalmol. 2014 Dec;92(8):e621-9.
Klettner et al.	Effect of long-term inflammation on viability and function of RPE cells.	Exp Eye Res. 2020 Nov;200:108214. doi: 10.1016/j.exer.2020.108214.
Kohno et al.	Photoreceptor proteins initiate microglial activation via Toll-like receptor 4 in retinal degeneration mediated by all-trans-retinal.	J Biol Chem. 2013 May 24;288(21):15326-41.
Kindzelskii et al.	Toll-Like Receptor 4 (TLR4) of Retinal Pigment Epithelial Cells Participates in Transmembrane Signaling in Response to Photoreceptor Outer Segments	J Gen Physiol . 2004 Aug;124(2):139-49.
Lewin	Geographic atrophy in age-related macular degeneration and TLR3.	N Engl J Med. 2009 May 21;360(21):2251; author reply 2255-6.
Liew et al.	Geographic atrophy in age-related macular degeneration and TLR3.	N Engl J Med. 2009 May 21;360(21):2252; author reply 2255-6.
Ling et al.	Associations of TLR4 gene polymorphisms with the risk of age-related macular degeneration in a Chinese Han population.	Medicine (Baltimore). 2019 May;98(19):e15583.
Liu et al.	Toll-like receptor 4 gene polymorphisms rs4986790 and rs4986791 and age-related macular degeneration susceptibility: a meta-analysis.	Ophthalmic Genet. 2020 Feb;41(1):31-35.
Ma et al.	Association of toll-like receptor 3 polymorphism rs3775291 with age-related macular degeneration: a systematic review and meta-analysis.	Sci Rep. 2016 Jan 22;6:19718.
Maloney et al.	Choroidal neovascular membranes express toll-like receptor 3.	Jr. Ophthalmic Res. 2010;44(4):237-41.

Mulfaul et al.	Toll-Like Receptors and Age-Related Macular Degeneration.	Adv Exp Med Biol. 2018;1074:19-28.
Mulfaul et al.	Toll-like Receptor 2 Facilitates Oxidative Damage-Induced Retinal Degeneration.	Cell Rep. 2020 Feb 18;30(7):2209-2224.e5.
Murakami et al.	Programmed necrosis, not apoptosis, is a key mediator of cell loss and DAMP-mediated inflammation in dsRNA-induced retinal degeneration	Cell Death Differ . 2014 Feb;21(2):270-7.
Paimela et al.	The effect of 17beta-estradiol on IL-6 secretion and NF-kappaB DNA-binding activity in human retinal pigment epithelial cells.	Immunol Lett. 2007 Jun 15;110(2):139-44.
Patel et al.	Toll-like receptor 3 (TLR3) protects retinal pigmented epithelium (RPE) cells from oxidative stress through a STAT3-dependent mechanism.	Mol Immunol. 2013 Jun;54(2):122-31.
Patel et al.	A novel protective role for the innate immunity Toll-Like Receptor 3 (TLR3) in the retina via Stat3.	Mol Cell Neurosci. 2014 Nov;63:38-48.
Saeed et al.	The oxidative stress product carboxyethylpyrrole potentiates TLR2/TLR1 inflammatory signaling in macrophages.	PLoS One. 2014 Sep 3;9(9):e106421
Sharma et al.	Does toll-like receptor-3 (TLR-3) have any role in Indian AMD phenotype?	Mol Cell Biochem. 2014 Aug;393(1-2):1-8.
Shiose et al.	Toll-like receptor 3 is required for development of retinopathy caused by impaired all-trans-retinal clearance in mice.	J Biol Chem. 2011 Apr 29;286(17):15543-55.
Sng et al.	Toll-like receptor 3 polymorphism rs3775291 is not associated with choroidal neovascularization or polypoidal choroidal vasculopathy in Chinese subjects.	Ophthalmic Res. 2011;45(4):191-6.
Stewart et al.	Expression of Toll-like receptors	Exp Eye Res. 2015

	in human retinal and choroidal vascular endothelial cells.	Sep;138:114-23.
Terheyden etal.	Basolateral activation with TLR agonists induces polarized cytokine release and reduces barrier function in RPE in vitro	Graefes Arch Clin Exp Ophthalmol . 2021 Feb;259(2):413-424
Wang et al.	Detection and biological activities of carboxyethylpyrrole ethanolamine phospholipids (CEP-EPs).	Chem Res Toxicol. 2014 Dec 15;27(12):2015-22.
Will-Orrego et al.	Amount of Mononuclear Phagocyte Infiltrate Does Not Predict Area of Experimental Choroidal Neovascularization (CNV).	J Ocul Pharmacol Ther. 2018 Sep;34(7):489-499.
Wörnle et al.	Inhibition of TLR3-mediated proinflammatory effects by Alkylphosphocholines in human retinal pigment epithelial cells.	Invest Ophthalmol Vis Sci. 2011 Aug 17;52(9):6536-44
Yang et al.	IL-10 is significantly involved in HSP70-regulation of experimental subretinal fibrosis.	PLoS One. 2013 Dec 20;8(12):e80288.
Yang et al.	Toll-like receptor 3 and geographic atrophy in age-related macular degeneration.	N Engl J Med. 2008 Oct 2;359(14):1456-63.
Zareparsi et al.	Toll-like receptor 4 variant D299G is associated with susceptibility to age-related macular degeneration.	Hum Mol Genet. 2005 Jun 1;14(11):1449-55.
Zhou et al.	Association study of toll-like receptors 4 polymorphisms and the risk of age-related macular degeneration: a meta-analysis.	Ophthalmic Genet. 2020 Dec;41(6):579-584.
Zhou et al.	Toll-like receptor 3 C1234T may protect against geographic atrophy through decreased dsRNA binding capacity.	FASEB J. 2011 Oct;25(10):3489-95.
Zhu et al.	Increase in peripheral blood mononuclear cell Toll-like receptor 2/3 expression and reactivity to their ligands in a	Mol Vis. 2013 Aug 6;19:1826-33

	cohort of patients with wet age-related macular degeneration.	
Zhu et al..	C5a and toll-like receptor 4 crosstalk in retinal pigment epithelial cells.	Mol Vis. 2015 Sep 29;21:1122-9.
